# Postural stability measures in healthy miniature Dachshunds obtained using a pressure mat and a force platform: a validity and reliability study

**DOI:** 10.1186/s12917-023-03633-0

**Published:** 2023-06-26

**Authors:** Aliah F. Shaheen, Daniela Lins, Thais Toledo, Constanza B. Gómez Álvarez

**Affiliations:** 1 Department of Life Sciences, Brunel University London, London, UK; 2 Department of Mechanical Engineering Sciences, University of Surrey, Guildford, UK; 3 Faculty of Agronomy and Veterinary Medicine, University of Brasilia, Brasilia, Brazil; 4 Faculty of Veterinary Medicine, Federal University of Mato Grosso, Cuiabá, Brazil; 5 Department of Veterinary Medicine, University of Cambridge, Cambridge, UK

**Keywords:** Validation, Reliability, Postural stability, Postural sway, Sway path, CoP, Centre of pressure, Canine, Miniature dachshunds

## Abstract

**Background:**

Miniature Dachshunds have a high prevalence of neurological and musculoskeletal diseases potentially affecting their balance. The postural stability of dogs in quiet standing is an indicator of postural control and can aid in diagnosing and monitoring lameness and other pathologies affecting balance. Measures of centre of pressure (CoP) can be obtained from force and pressure platform systems to evaluate postural stability, however the two systems have not been compared and the latter has not been validated in dogs. The aims of this study were to assess the validity and reliability of using a pressure mat compared to a force platform and report normative values of CoP measures in healthy miniature Dachshunds. Forty two healthy miniature Dachshunds of smooth, long and wire-haired breed types stood still on a pressure mat (Tekscan MatScan®) placed on a force platform and the two systems were synchronised. Maximum anterior-posterior (AP) and medial-lateral (ML) ranges, sway path and 95% area of a best-fit ellipse were computed. Bland-Altman plots and coefficients of correlation assessed validity; intra-class correlation coefficients (ICC) assessed inter-test reliability for both systems. Non-linear regression analyses were used to describe the relationship between CoP and demographic measures.

**Results:**

Strong correlations for AP range, ML range and 95% ellipse area and moderate correlation for sway path were found between the two devices. ICC showed good reliability (0.75–0.90) for AP range and moderate (0.5–0.75) for ML range and the 95% ellipse area for both devices. Sway path reliability was excellent (> 0.90) with the force platform but moderate with the pressure mat. Age was positively correlated with balance (inversely correlated with all measures except sway path), while weight explained 94% (force platform) and 27% (pressure mat) of the variance in sway path.

**Conclusions:**

Pressure mats can be used to obtain valid and reliable measures of CoP and replace use of force platforms. Older (non-senior) and heavier (non-obese) dogs show better postural stability. Clinical examinations should include the use of a range of CoP measures when assessing postural balance, while accounting for the effects of age and body weight.

## Introduction

Interest in studying postural control and stability of dogs date back to the early 1900s [1]. Studying postural stability can reveal important information about the biological mechanisms responsible for postural control [2] and the responses to visual and other perturbations [3]. More recently, measures of postural stability have been used clinically to diagnose lameness in dogs [4, 5, 6] and ponies [7], and to measure the effects of ageing on postural control using adults and senior dogs [8].

Postural stability can be assessed during quiet standing using measures of the centre of pressure (CoP) in bipeds [9] and quadrupeds [4]. CoP is the point at which the ground reaction force is applied. Movements of the CoP are related to accelerations of the body’s centre of mass and are how movements of the centre of mass are controlled [10]. Force platforms are considered to be the gold standard measurement tool in human medicine to obtain measures of CoP and their reliability have been assessed in multiple studies [11, 12, 9]. Previous studies have used metrics obtained from the movements of the CoP (position, velocity and acceleration) in studies concerned with the assessment of balance [13].

Pressure mat systems such as Tekscan MatScan® are portable and easy to use, making them a preferred measurement tool in clinical settings. Recently, these systems have been used to obtain CoP measures in quiet standing in humans [14, 15]. However, there is limited evidence of their validity in obtaining these measures, with only a single study testing its validity against force platforms in humans [16] and a preliminary study in lame dogs [4]. The main difference between the two measurement systems, is that whilst force platforms measure the three components of the ground reaction force, pressure mats typically only measure the vertical component. Therefore, it is important that measurements of CoP obtained from pressure systems are assessed for their validity and reliability before their use in clinical studies.

The aim of this study was to assess the validity and reliability of obtaining CoP measures of postural stability in sound dogs using a pressure mat (Tekscan MatScan®) as compared to force platform measurements. A secondary aim was to collect normative measures of CoP from a non-lame group of miniature Dachshunds for use of future studies that include clinical populations. This is a population of clinical interest because of their high prevalence of spinal problems.

## Methods

A sample size calculation using G*Power 3.1.9.7, with α level of 0.05, a power of 0.8, effect size of 0.4, and based on a correlation analysis suggested that a minimum of 34 dogs were needed for the analysis. Forty two healthy miniature Dachshunds dogs (30 females) of different breed type (16 smooth-haired, 16 long-haired and 10 wire-haired) participated in the study after being recruited through the Dachshund Breed Council UK. Dogs had to be above 6 months of age to be included and they were excluded if they had an existing problem that could cause pain, lameness or postural imbalance.

Owners completed a health form for their dogs and the dogs were examined by a veterinary surgeon blinded to the information provided by the owners. Dogs were found to be free of lameness or other signs of pain based on a visual lameness assessment by the veterinary surgeon and confirmed by force platform data of their gait. The recruited dogs had a mean age of 4.3 (± 3.2) years old, weight of 5.4 (± 1.4) kg, Body Condition Score (BCS) of 3.4 (± 0.9) out 5 [17], length of 26.9 (± 2.8) cm, height at the withers of 19.2 (± 1.9) and a length-to-height ratio of 1.40 (± 0.11).

**Fig. 1:**
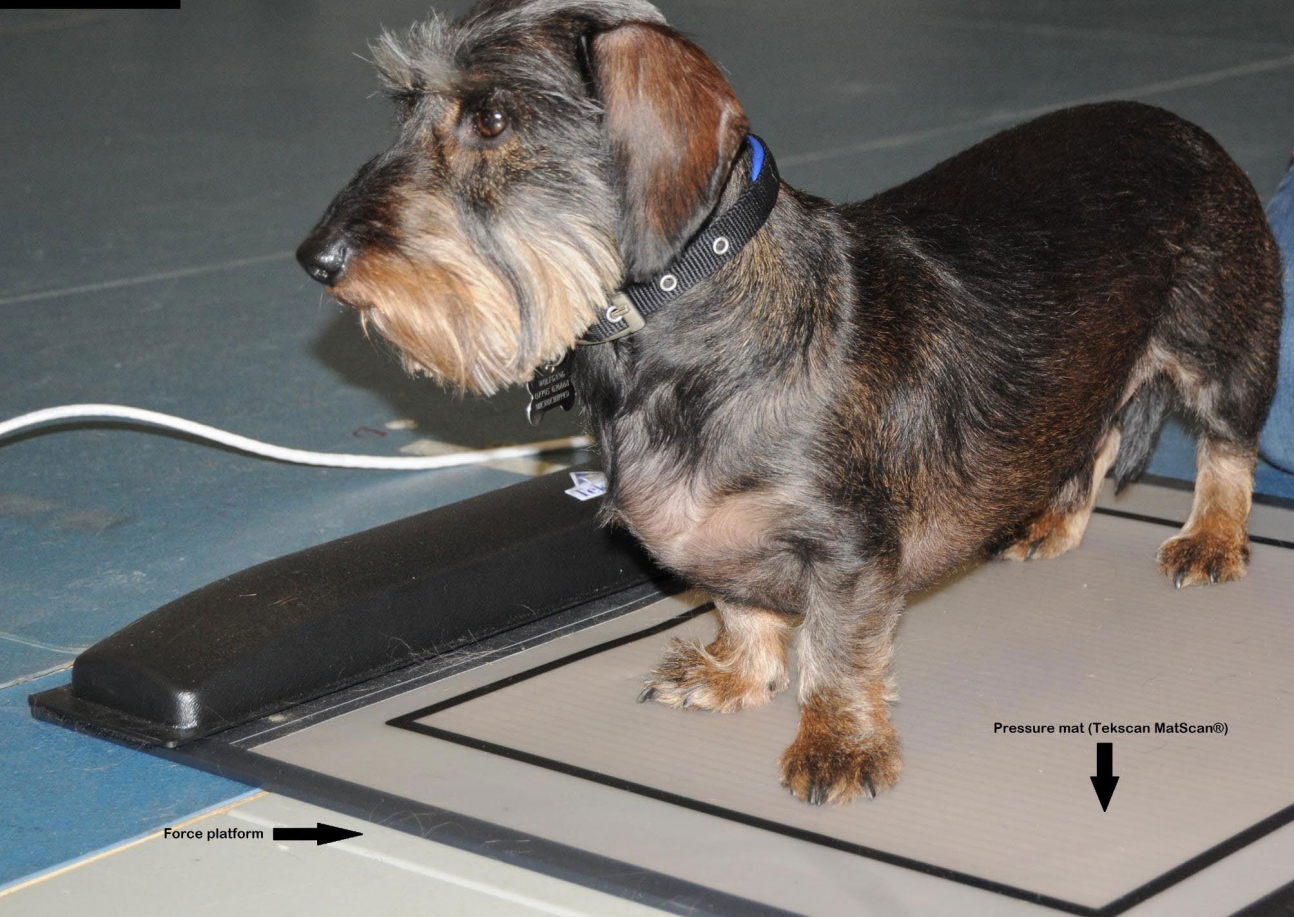
One of the dogs in quiet standing during a measurement on a synchronised pressure mat (Tekscan Matscan®) on top of a force platform

Measurement trials of the dogs in quiet standing were obtained in the presence of their owners by one of the researchers (AS) in the Human Movement Laboratory of the University of Surrey, as the dogs stood still on a pressure mat (MatScan®, Tekscan, South Boston MA, USA) running at 60 Hz placed on top of a force platform (AMTI, Watertown MA, USA) running at 100 Hz. The two systems were synchronised and calibrated as per the instructions of the manufacturers, the force platform was zeroed at the start of the session and the pressure mat was calibrated using the body weight (50 kg) of one of researchers at the start of each session. Five standing trials of 10 s durations were collected. Trials where the dog was seen to move the body or the head, moved its tail, unloaded one of its limbs or attempted to sit were discarded before analysis. Figure 1 shows one of the dogs in quiet standing during a measurement.

Measures of CoP were computed from the data obtained using the two measurement systems, these measures were; maximum anterior-posterior (AP) range, maximum medial-lateral (ML) range, sway path and 95% area of a best-fit ellipse. Data from the pressure mat was resampled to 100 Hz and a re-analysis of these measures was conducted and compared to data obtained using the original frequency of 60 Hz.

Bland-Altman plots and Pearson’s coefficient of correlation were used to assess the validity of the CoP measures obtained using the pressure mat compared to those obtained using the force platform. Intra-class correlation coefficients (ICC) were used to assess the inter-test reliability of the CoP measures obtained using the two devices [18]. ICC estimates and their 95% confidence intervals were calculated based on a mean-rating (k = 5), absolute agreement, 2-way mixed effects model [19].

ANOVA tests were used to investigate differences in age, weight, height, length-to-height ratio and BCS between males and females and between the three breed types (smooth-haired, long-haired and wire-haired) and Pearson’s correlations were used to look at relationships between these demographic measures. ANOVA tests were also used to investigate differences in the CoP measures obtained using both devices with sex and breed type as independent factors and age, weight, height, length-to-height ratios and BCS used as covariates. Linear and non-linear regression (logarithmic, quadratic, exponential, power) analysis was used to assess the relationship between CoP measures and demographic measures (age, weight, height, BCS and length-to height ratio).

SPSS statistical package version 26 (SPSS Inc, Chicago, IL) was used to run all statistical tests.

This study has been checked against the ARRIVE 2.0 Essential 10 list.

## Results

### Validity and reliability of CoP measures obtained using a pressure mat (***Tekscan Matscan®)***

**Fig. 2:**
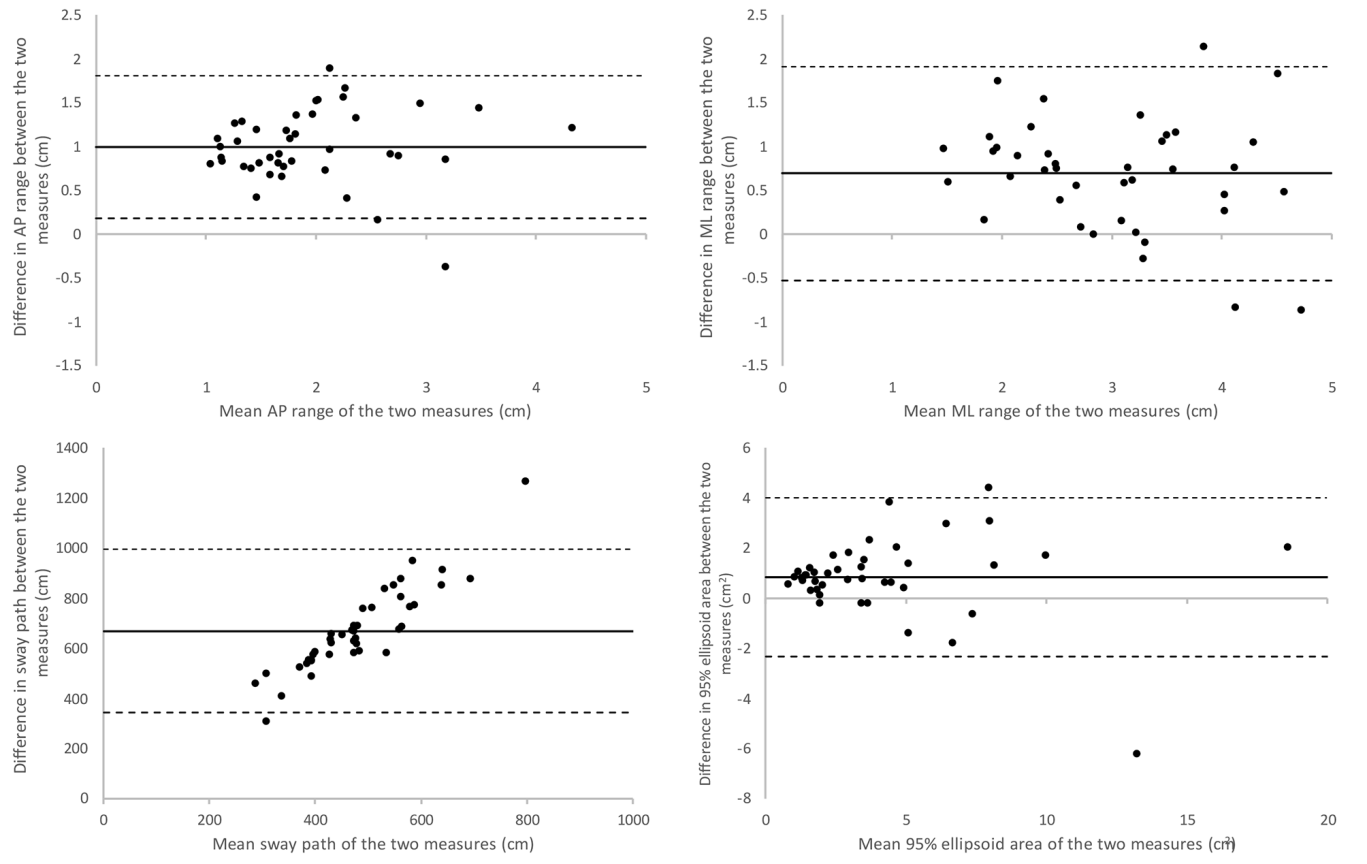
Bland-Altman plots showing the difference in the centre of pressure (CoP) measures (Anterior-Posterior AP range, Medial-Lateral ML range, 95% area of best-fit ellipse and sway path) between the force platform and pressure mat (Tekscan Matscan®). A positive bias indicates a greater mean for the measure obtained from the force platform

Bland-Altman plots (Fig. 2) show a good level of agreement between the two devices for AP range, ML range and the 95% ellipse area but a large difference in sway path. There is a positive bias for all measures, with greater values for measures obtained from force platforms. This difference is relatively small with relatively narrow agreement limits for AP range (0.99 ± 0.41 cm), ML range (0.69 ± 0.62 cm) and the 95% ellipse area (0.83 ± 1.61 cm^2^), but is quite large for sway path (670 ± 167 cm). There is also an obvious trend in the sway path measure that suggests that the difference between the measures increases with an increase in the mean. The resampling of the pressure mat data to 100 Hz resulted in a negative effect on the correlations between the devices and therefore the data with the original sampling frequency are presented here. The correlation analyses showed that there was a strong correlation for AP range, ML range and 95% ellipse area, but only moderate correlation for sway path between the two devices as shown in Table 1.

**Table 1:** Means and standard deviations of centre of pressure (CoP) measures obtained using a force platform and a pressure mat; and Pearson correlation coefficients between the two systems and their p-values

CoP Measure	Force Platform	Pressure Mat	Pearson Correlation	
	Mean (SD)	Mean (SD)	**R**	p-value
**Anterior-posterior (AP) range (cm)**	2.45 (0.75)	1.46 (0.74)	**0.843**	**< 0.001**
**Medial-lateral (ML) range (cm)**	3.45 (0.98)	2.76 (1.07)	**0.816**	**< 0.001**
**95% area of best-fit ellipse (cm** ^**2**^ **)**	4.64 (3.56)	3.80 (3.70)	**0.900**	**< 0.001**
**Sway path (cm)**	816.38 (186.61)	146.78 (44.89)	**0.490**	**0.001**

The ICC reliability of the two measurement systems was similar for the AP range, ML range and the 95% ellipse area, with both systems showing a good reliability (between 0.75 and 0.90) for AP range and a moderate reliability (between 0.5 and 0.75) for the ML range and 95% ellipse area. The reliability of the sway path measure was excellent (above 0.90) when obtained from the force platform but only moderate with the pressure mat measures (Table 2).

**Table 2:** Intra class coefficient (ICC) values and the lower and upper bound of the 95% confidence interval for the centre of pressure (CoP) measures obtained using a force platform and a pressure mat. AP (anterior-posterior), ML (medial-lateral)

CoP Measure	Force Platform			Pressure Mat		
	ICC	95% confidence interval		ICC	95% confidence interval	
		Lower bound	Upper bound		Lower bound	Upper bound
**AP range**	**0.792**	0.657	0.885	**0.768**	0.681	0.871
**ML range**	**0.704**	0.510	0.837	**0.618**	0.368	0.788
**95% area of best-fit ellipse**	**0.707**	0.519	0.836	**0.726**	0.548	0.848
**Sway path**	**0.986**	0.977	0.992	**0.718**	0.534	0.844

### Normative CoP measures for healthy miniature dachshunds

The results show that there was a difference in height and weight between sex, with males having a greater weight (p = 0.011, 3.54±1.0 kg vs. 3.41±0.90 kg) and height (p = 0.003, 20.50±2.62 cm vs. 18.64±1.15 cm). For breed type, there was a difference between the groups in age (p = 0.021) and height (p = 0.017), where the smooth-haired dogs were older and taller than dogs in the other two groups (Table 3). Therefore, any differences in CoP measures between breed types would need to be interpreted in light of an existing difference in these measures.

**Table 3:** Showing the means for the demographic measures and the computed centre of pressure (CoP) measures obtained using the force platform and the pressure platform Tekscan Matscan® for males and females, and the different breed types (smooth, long and wire-haired) and the results of the ANOVA tests, significance set at p < 0.05. AP (anterior-posterior), ML (medial-lateral). *p < 0.05, **p < 0.01, ***p < 0.001

	Sex			Breed type			
	Males	Females	p-value	Smooth-haired	Long-haired	Wire-haired	p-value
**Age (years)**	2.98 (3.83)	4.72 (3.14)	0.169	3.20 (3.25)	6.17 (3.19)	3.23 (2.42)	0.021*
**Weight (kg)**	3.54 (1.01)	3.41 (0.90)	0.011*	5.67 (1.07)	5.43 (1.87)	4.88 (0.94)	0.374
**Height at the withers (cm)**	20.5 (2.62)	18.64 (1.15)	0.003**	20.22 (2.28)	18.46 (1.01)	18.60 (1.54)	0.017*
**length-to-height ratio**	1.42 (0.15)	1.40 (0.11)	0.693	1.38 (0.12)	1.46 (0.08)	1.37 (0.14)	0.088
**Body condition score - BCS**	3.54 (1.01)	3.41 (0.90)	0.687	3.56 (0.85)	3.64 (1.08)	3.00 (0.71)	0.204
**CoP Measure**	**Force Platform (AMTI)**						
**AP range (cm)**	2.91 (1.11)	2.27 (0.46)	0.117	2.74 (0.84)	2.22 (0.66)	2.38 (0.66)	0.246
**ML range (cm)**	3.83 (1.41)	3.30 (0.73)	0.254	3.71 (1.16)	3.16 (0.75)	3.48 (0.99)	0.347
**95% area of best-fit ellipse (cm** ^**2**^ **)**	7.01 (5.27)	3.69 (2.02)	0.310	6.37 (4.71)	3.12 (1.79)	4.29 (3.56)	0.089
**Sway path (cm)**	753 (191)	842 (182)	0.026*	758 (138)	848 (236)	860 (155)	0.103
**CoP Measure**	**Pressure platform (Tekscan Matscan®)**						
**AP range (cm)**	1.95 (0.90)	1.26 (0.57)	0.179	1.78 (0.88)	1.27 (0.57)	1.26 (0.61)	0.367
**ML range (cm)**	3.23 (1.32)	2.57 (0.91)	0.158	2.93 (1.22)	2.68 (0.90)	2.61 (1.14)	0.904
**95% area of best-fit ellipse (cm** ^**2**^ **)**	5.74 (4.64)	3.02 (3.00)	0.688	5.36 (5.06)	2.67 (2.13)	3.11 (2.25)	0.387
**Sway path (cm)**	137 (57)	151 (40)	0.166	126 (39)	170 (45)	142 (39)	0.006**

There were no differences between males and females and between different breed types for all CoP measures obtained with both measurements systems with the exception of sway path (Table 3). The results for sway path were inconsistent between the force platform measurements and the pressure mat. Force platform measures suggested that females had lower sway path values than males (adjusted p = 0.026) but this was not shown by the measures obtained by the pressure mat (adjusted p = 0.103). On the other hand, the pressure platform mat measures for CoP sway path suggested a difference by breed type (adjusted p = 0.006), whilst force platform CoP sway path measures did not show a difference (adjusted p = 0.166).

**Fig. 3:**
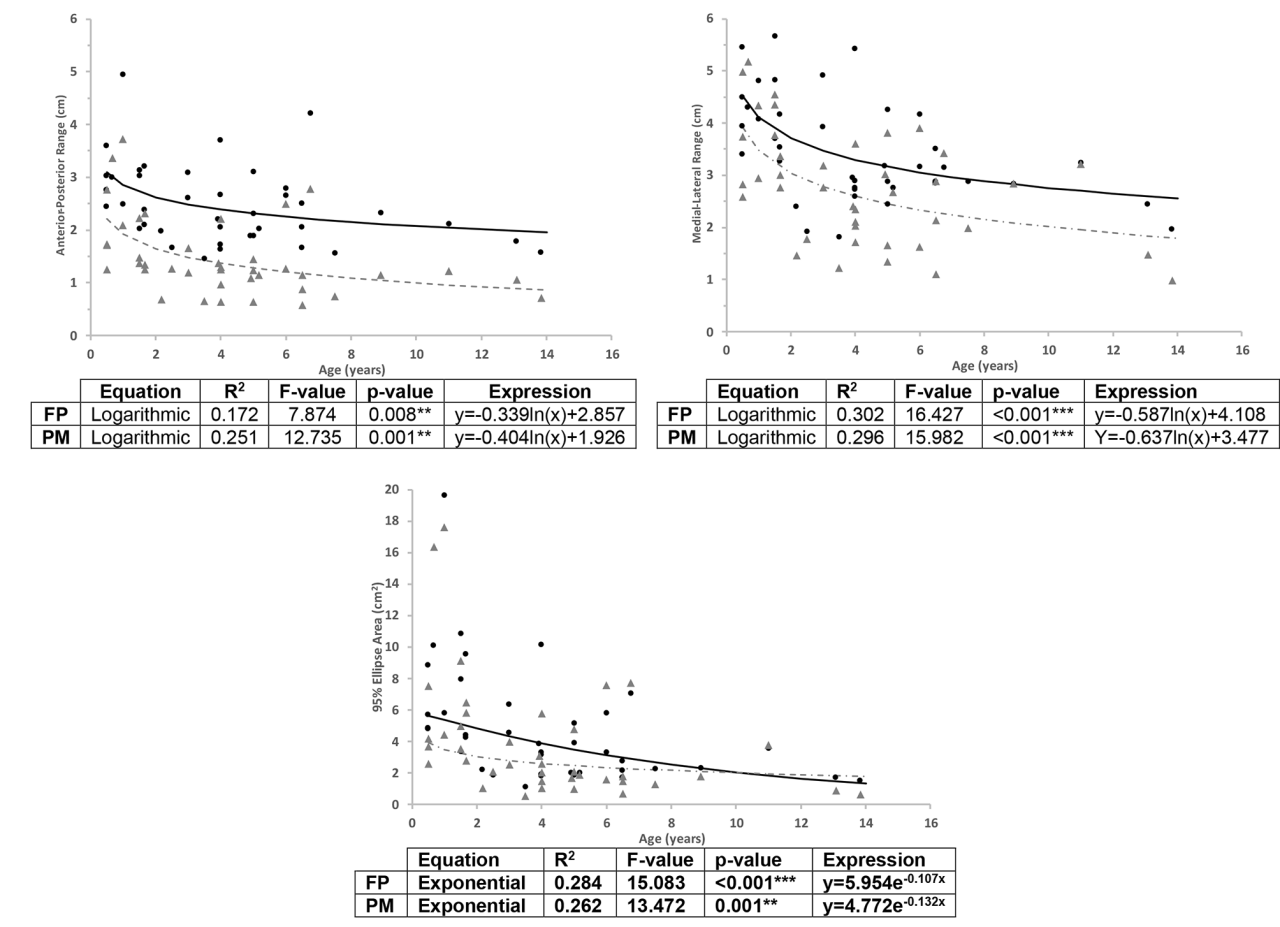
Regression equations showing the relationship between Anterior-Posterior range, Medial-Lateral range, 95% ellipse area and age for the CoP measures obtained using the force platform (FP) (black circles and continuous black line) and pressure mat (Tekscan Matscan®) (PM) (grey triangles and dashed line)

**Fig. 4:**
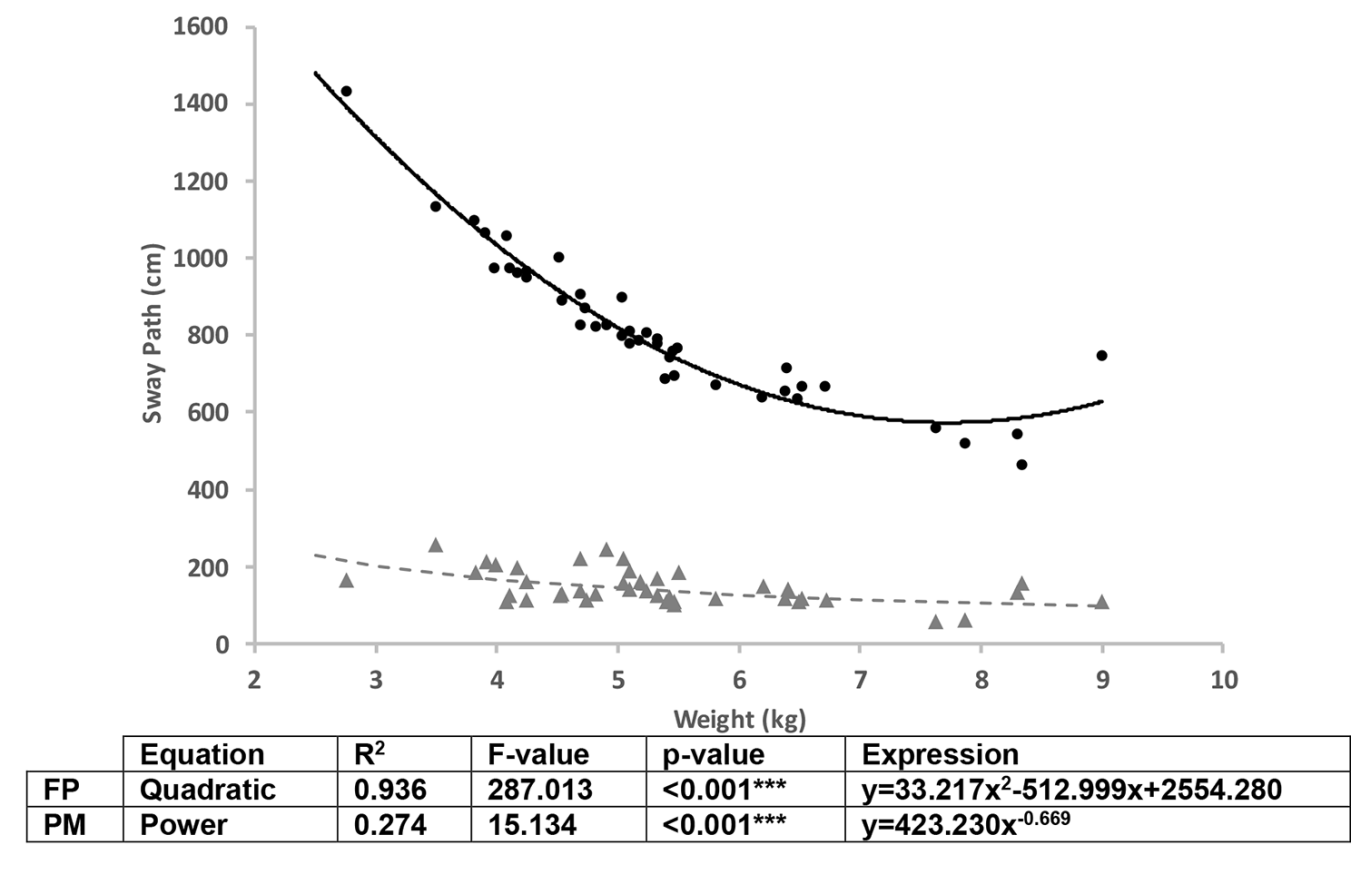
Regression equations showing the relationship between sway path and weight for the CoP measure obtained using the force platform (black circles and continuous line) and pressure mat (Tekscan Matscan®) (grey triangles and dashed line)

Correlational analysis between the demographic measures showed that age and weight are independent variables, but that height (r = 0.582, p = < 0.001) and BCS (r = 0.363, p = 0.018) were correlated with weight. Regression analysis showed that there were relationships between age and the AP range, ML range and 95% Ellipse area measures obtained by both devices. These relationships were best described using non-linear equations (see Fig. 3). An increase in age resulted in a decrease in these three measures but had no effect on sway path. Suggesting that older adult dogs were more stable. Interestingly, weight had a relationship with sway path but not with the other CoP measures, where an increase in weight resulted in a reduced sway (Fig. 4). Weight was found to explain 94% of the variance in sway path measured by the force platform (p < 0.001, using a quadratic regression model) and 27% in sway path measured by the pressure mat (p < 0.001, using a power regression model), these relationships and the expressions are shown in Fig. 4.

## Discussion

The study aimed to assess the validity and reliability of postural stability parameters measured with the pressure mat (Tekscan Matscan®) compared to the force platform - commonly used to obtain CoP measures - as well as to present a normative database for CoP measures of miniature Dachshunds from both systems which can be used in future canine studies with patient populations.

The results showed that CoP measures obtained from the pressure mat system have good agreement and excellent correlations with those obtained using the force platform, with the exception of sway path. Sway path measures were different in magnitude and there was only a good correlation between the two systems. The large difference seen in the sway path measure is expected because sway path is largely dependent on the frequency of capture, which was different for the two devices. Although the difference in the frequencies of capture may go some way in explaining this difference, it does not seem to be the only cause; resampling of the pressure data to the same frequency did not improve the correlations. It is therefore likely that differences in sway path were not just due to the frequency of capture but are also related to inherent differences in the sensitivity of the devices and the type of data obtained (3D for force platform with CoP accuracy of < 0.5 mm vs. vertical force for pressure mat of a measuring resolution of 1.4 sensel/cm^2^ and a range of 34.5–86.2 N/cm^2^).

Both devices had comparable reliabilities (as assessed using ICC) for CoP measures except for sway path. This measure when obtained using a force platform has an excellent between-trial reliability, whilst all other measures (including sway path measured by the pressure mat) had moderate or good reliabilities between trials. Whilst the study showed comparable reliabilities of the two systems, one advantage of using the pressure system is that it allows the researcher to easily spot when the animal off-loads one of the limbs which would have a considerable effect on CoP measures. This is possible because the system provides measures of the force cells loaded by each paw, whilst a force platform only provides a measure of the movement of the overall ground reaction force vector, making these measurement errors more difficult to spot.

Sway path was the only measure that was found to be positively related to the dog’s weight. Regression equations used to describe this relationship showed that weight can explain 94% of the difference in sway path when obtained using the force platform and 27% when obtained using the pressure mat. The results are in line with findings in horses [20] and humans [21], and with a study on dogs [8] where weight and height are found to be positively correlated with stability when participants are not obese, as in our study. One way to reduce the effect of this on clinical interpretation is to normalise the measure to the weight of the animal, this has also been suggested by Chiari et al. [21].

The other CoP measures had significant relationships with age. Regression analysis show that age can explain between 17 and 30% of the difference in these CoP measures (using non-linear equations). Similarly, age was positively correlated with stability (reduction in AP range, ML range and 95% Ellipse area). The positive relationships between stability (as assessed by CoP measures) and age may appear to contradict the current knowledge based on human research that suggests that CoP measures are sensitive to changes in stability caused by age, such as deficits in vision, proprioception or muscle strength [9] and based on senior dogs defined as being older than 75% of their lifespan [8]. In the latter study, some of the tested senior dogs also presented with a degree of joint pain. It is important to note that in our study, we used a healthy adult population, not necessarily senior dogs, and excluded dogs with known lameness or pain. Furthermore, the study did not aim to study the effect of senior age on CoP measures and stability.

The results showed that on the whole, there were very little to no differences in the CoP measures between males and females and between the breed types. Where there were differences, these were only found in sway path and were inconsistent between the two measurement systems.

Finally, there were a number of limitations to data presented in this study. Firstly, the CoP measures reported here are based on standing on the pressure mat directly with no cover. Previous canine studies have demonstrated that ground reaction force data is affected by the surface [22, 23] and human studies have also suggested that a different balancing mechanism is employed on different surfaces [24]. Therefore, this should be taken into account when using the normative data from this study. Another limitation of the study is related to the length of the trials. Measurement trials were kept to 10 s only for convenience, this is similar to the study by Manera et al. [6] but is shorter than the 20 s used in other canine studies [4, 5]. Future canine studies should assess the effect of trial length on the reliability of the CoP measures in order to reach a consensus within the scientific community.

## Conclusions

Pressure mat systems can be used to obtain valid and reliable measures of CoP and can replace the use of force platforms. However, care should be taken in interpreting differences between populations where these measurements are not obtained using the same technology, this is particularly true for sway path (or measures derived from sway path). It is important to include a range of CoP measures in clinical assessments when evaluating balance in dogs, and comparisons to healthy/normative values should account for expected variations caused by age and weight.

## Data Availability

The datasets used during the current study are available from the corresponding author on reasonable request.
